# Development of a fluorescence-based method for monitoring glucose catabolism and its potential use in a biomass hydrolysis assay

**DOI:** 10.1186/1754-6834-1-17

**Published:** 2008-11-19

**Authors:** Lisa J Haney, James G Coors, Aaron J Lorenz, D Raj Raman, Robert P Anex, M Paul Scott

**Affiliations:** 1 Syngenta Seeds Inc, Bloomington, IL, USA; 2 Department of Agronomy, University of Wisconsin-Madison, Madison, WI, USA; 3 Department of Agricultural and Biosystems Engineering, Iowa State University, Ames, IA, USA; 4 Corn Insects and Crop Genetics Research Unit, ARS, USDA, USA

## Abstract

**Background:**

The availability and low cost of lignocellulosic biomass has caused tremendous interest in the bioconversion of this feedstock into liquid fuels. One measure of the economic viability of the bioconversion process is the ease with which a particular feedstock is hydrolyzed and fermented. Because monitoring the analytes in hydrolysis and fermentation experiments is time consuming, the objective of this study was to develop a rapid fluorescence-based method to monitor sugar production during biomass hydrolysis, and to demonstrate its application in monitoring corn stover hydrolysis.

**Results:**

Hydrolytic enzymes were used in conjunction with *Escherichia coli *strain CA8404 (a hexose and pentose-consuming strain), modified to produce green fluorescent protein (GFP). The combination of hydrolytic enzymes and a sugar-consuming organism minimizes feedback inhibition of the hydrolytic enzymes. We observed that culture growth rate as measured by change in culture turbidity is proportional to GFP fluorescence and total growth and growth rate depends upon how much sugar is present at inoculation. Furthermore, it was possible to monitor the course of enzymatic hydrolysis in near real-time, though there are instrumentation challenges in doing this.

**Conclusion:**

We found that instantaneous fluorescence is proportional to the bacterial growth rate. As growth rate is limited by the availability of sugar, the integral of fluorescence is proportional to the amount of sugar consumed by the microbe. We demonstrate that corn stover varieties can be differentiated based on sugar yields in enzymatic hydrolysis reactions using post-hydrolysis fluorescence measurements. Also, it may be possible to monitor fluorescence in real-time during hydrolysis to compare different hydrolysis protocols.

## Background

Fermentation reactions are important for the production of many valuable products including pharmaceuticals, beverages, and biofuels. In lignocellulosic ethanol production, biomass feedstocks are chemically pretreated, hydrolyzed by cellulases and hemicellulases, and the resulting sugars are fermented by yeast or bacteria to produce ethanol [1]. In large-scale production of biocommodities such as ethanol, feedstocks have a large and often dominant impact on process economics and process development [2]. For example, the amount of sugar available to fermentation reactions is important because in the absence of limiting factors, substrate availability determines product yield; thus methods to measure sugar available to a fermentation reaction are potentially valuable for selecting feedstocks.

One approach is to complete a simultaneous saccharification and fermentation (SSF) process [3] and to assay residual sugars and inhibitory products such as glucose, cellobiose, and acetic acid by high performance liquid chromatography (HPLC), and ethanol concentration by gas chromatograph (GC) or HPLC. The whole procedure takes about 168 hours according to the SSF protocol specified by the National Renewable Energy Laboratory (NREL) [4]. Recently, Weimer et al have developed a higher throughput method to predict the fermentability of cellulosic biomass to ethanol through *in vitro *gas production [5]. In this procedure, fermentations are carried out in sealed serum bottles, and the gas produced is measured as an indicator of the digestibility of the cellulosic biomass.

In contrast to full SSF processes, many sugar detection and quantitation methods can be employed, including chemical reducing sugar assays and enzymatic assays [6,7]. All of these methods require sampling the fermentation reaction and measuring sugars in the sample. However, because cellulases and hemicellulases are product inhibited, simple approaches that only involve hydrolytic enzymes and a sugar assay (or biosensor) yield low, non-representative estimates of conversion potential. Determining a meaningful sugar yield requires that sugars be removed as they are produced, as is done in SSF processes [3].

Once a sugar scavenger has been added to the mixture of hydrolytic enzymes, the problem of product inhibition has been solved, but another problem replaces it: the sugars produced are consumed as they are produced, making measurements of sugar concentrations a poor predictor of total sugar released. For this reason, simply relying on a sugar assay or a sugar biosensor, such as one described by Lidgren et al [8], will not work. Therefore, our objective was to develop a system that could be used to monitor glucose catabolism as an indicator of feedstock convertibility and to demonstrate its application to monitoring corn stover hydrolysis in a process similar to that used for lignocellulosic ethanol production. Such a system would be useful for rapidly screening varieties for suitability as biomass feedstocks in plant breeding programs and for evaluating different hydrolytic systems. Furthermore, this system could be used to measure sugar production in a wide range of other experiments.

## Methods

### Overview

Our approach was to develop a microbial system that reports sugar levels in a reaction that mimics the NREL lignocellulosic ethanol production process [4]. The presence of microbes in this system overcomes the feedback inhibition problems associated with enzyme-only methods. *Escherichia coli *strain CA8404 was selected as the microbe because it carries the crp* mutation which reduces catabolite repression and thereby allows both the 5- and 6-carbon sugars produced from corn stover hydrolysis reactions to be metabolized simultaneously [9]. As the *E. coli *metabolize the sugars from the hydrolysis reaction, cell mass (*X*) changes at a rate of *dX/dt*. This change in culture cell mass can be monitored by light-scattering measurements of culture turbidity. However, certain reactions, including lignocellulosic biomass hydrolysis, produce substances that interfere with light-scattering measurements. In order to more easily monitor change in cell culture mass, the *E. coli *strain CA8404 was modified to produce a visual marker, green fluorescent protein (GFP) [10,11]. The sharp emission peak and specific wavelength requirement for excitation allow for a much greater specificity of detection than does light scattering alone. The version of GFP used in this study (S65T) has a maximum excitation wavelength of 490 nm and a maximum emission wavelength of 510 nm [12].

### Site-directed mutagenesis and transformation

Site-directed mutagenesis was conducted according to the Stratagene product QuikChange II^® ^Site-Directed Mutagenesis kit in order to produce a pPNptGreen plasmid without a functional GFP fluorophore (Stratagene, La Jolla, CA). Approximately 300 bp into the GFP coding sequence, DNA encoding a glutamate residue (GAA) was changed to encode a stop codon (TAA). (Primers for mutagenesis: Forward: GATGACGGGAACTACAAGACACGTGCTTAAGTCAAGTTTGAAGG; Reverse: CCTTCAAACTTGACTTAAGCACGTGTCTTGTAGTTCCCGTCATC.) The new plasmid was designated pPNptOchre. The original pPNptGreen plasmid and the pPNptOchre plasmid without the functional GFP fluorophore were transformed separately into *E. coli *strain CA8404 to produce the two strains, crp*-gfp and crp*-gfp^-^.

### Preparation of corn stover samples

At grain maturity, cobs were removed from the corn plants and all corn stover samples were cut at approximately six inches above the soil by a forage chopper. Approximately 0.8 kg of sample (wet weight) at a moisture content of about 35% was collected from each plot and samples were dried at 55°C for one week. The material from each sample was ground by a hammermill with a 1 mm screen. We did not evaluate the distribution of different botanical tissues in the ground material; however, the ground material appeared uniform. To minimize the effect of a non-uniform distribution of biological material in the sample we used multiple subsamples in each experiment.

### Product inhibition of hydrolytic enzyme mixture

To characterize the product inhibition of the enzyme preparation Multifect^® ^A-40 (a cellulase/hemicellulase mixture from Genencor Intl.), we carried out hydrolysis reactions in the presence or absence of 10 mM d-glucose (CAS# 50-99-7, Sigma-Aldrich Inc., St. Louis, MO). Each treatment was run with four replicates using 5 mg of a stover sample treated with a 1:20 dilution of enzyme Multifect^® ^A-40 in citrate-phosphate buffer (21 ml 0.1 M citric acid and 29 ml 0.2 M sodium phosphate, in a final volume of 100 ml, pH 5.5). Hydrolysis was conducted at 60°C for 90 min. Following hydrolysis, the tubes were centrifuged for 1.5 min. at 10,000 × *g *in a microcentrifuge (Spectrafuge, Orem, UT). An aliquot of the supernatant from the hydrolysis reaction was measured with a hexokinase glucose assay kit (Sigma-Aldrich Inc., St. Louis, MO). The absorbance was measured at 340 nm (OD_340_) using the MRXII plate reader by DYNEX (Magellan Biosciences Company, Chelmsford, MA). The absorbance value was converted to glucose yield with a standard curve constructed by plotting OD_340 _values versus glucose concentrations following analysis of a series of solutions with known glucose concentrations.

### Growth of liquid cultures for growth characterization experiments

Cultures of *E. coli *crp*-gfp and crp*-gfp^- ^paired by treatment were grown in modified 1 × M9 minimal media [13]. The M9 media was modified by the addition of Kanamycin (50 μg/ml), thiamine (0.01% w/v), and ammonium chloride (5 mg/ml). Also, different carbon source concentrations were provided to the cultures than the carbon source described by Sambrook and Russell [13]. d-glucose (CAS# 50-99-7, Sigma-Aldrich Inc., St. Louis, MO), and d-xylose (CAS# 58-86-6, Sigma-Aldrich Inc., St. Louis, MO) solutions were made in the appropriate concentrations indicated in each experimental procedure below. All sugar solutions were filter-sterilized and frozen. Sugar mixtures were combined from separate, sterilized glucose and xylose sugar solutions. Cultures were grown in clear, 96-well cell culture plates (Product # 92096, Techno Plastic Products, Trasadingen, Switzerland) and sealed with AirPore™ seals (Qiagen, Valencia, CA) in order to ensure that enough oxygen was available to the cultures. The plates were then securely fastened down in the Innova 4300 incubator shaker (New Brunswick Scientific, Edison, New Jersey), and allowed to incubate with shaking at 37°C and 225 rpm. When it was time to take a measurement, the AirPore™ seal was removed only from the wells to be measured, and absorbance (OD_595_) measurements were taken by the MRXII plate reader (Dynex – a Magellan Biosciences Company, Chelmsford, MA). The samples from the wells to be measured were then transferred into a black, 96-well cell culture plate (Corning Incorporated Life Sciences, Lowell, MA), and fluorescence measurements (excitation wavelength: 485 nm, emission wavelength: 535 nm) were taken by the SpectraFluor Plus plate reader (Tecan US, Research Triangle Park, NC). Note that these wavelengths (595 nm for absorbance, 485 nm for excitation, and 535 nm for emission) were used consistently throughout the study. The AirPore™ seal was replaced on the clear 96-well plate and returned to the incubator. To obtain a value for GFP-specific fluorescence for each culture pair, the fluorescence reading of the crp*-gfp^- ^strain was subtracted from the fluorescence reading of the crp*-gfp strain.

### Characterization of the microbial system

#### Growth curves with different glucose concentrations

To establish whether it was possible to use GFP to detect differences in changes in culture cell mass in response to sugars, cultures of *E. coli *crp*-gfp and crp*-gfp^- ^were grown in modified 1 × M9 minimal media containing 2, 4, or 8 mg/ml d-glucose. Absorbance and fluorescence were measured every 2 h for 22 h, and the GFP-specific fluorescence was determined.

#### Sensitivity and dynamic range

To determine the sensitivity and dynamic range of the microbial system, cultures of *E. coli *crp*-gfp and crp*-gfp^- ^were grown in modified 1 × M9 minimal media containing d-glucose in concentrations ranging from 0.025 mg/ml to 6.0 mg/ml. Absorbance and fluorescence measurements were taken 20 h after inoculation, and the GFP-specific fluorescence was determined.

#### Glucose spiking

To determine the response time of the microbial system, glucose was added to the reaction when the culture reached stationary phase. Two sets of three replications of both *E. coli *strains crp*-gfp and crp*-gfp^- ^were grown in modified 1 × M9 minimal media containing 2 mg/ml d-glucose for 20 h. After 20 h, half of the cultures (one set) were randomly selected to receive an addition of 8 mg/ml d-glucose for a total of three replications each of spiked cultures and unspiked cultures. Absorbance and fluorescence were measured every 2 h, and the GFP-specific fluorescence was determined.

#### Stopping protein production

Another way we examined the response time of the microbial system was by stopping protein production when the culture was in mid-log phase. Six replications of both *E. coli *strains crp*-gfp and crp*-gfp^- ^were grown in modified 1 × M9 minimal media, containing 20 mg/ml d-glucose. Chloramphenicol was added to a random selection of half of the cultures after 13 h for a total of three replications each of cultures with chloramphenicol and without chloramphenicol. Absorbance and fluorescence were measured every hour, and the GFP-specific fluorescence was determined.

### Application of the microbial system

#### Post-hydrolysis monitoring

The microbial system described here, referred to as simultaneous saccharification and catabolism (SSC) was used to analyze corn stover samples of five different corn varieties. For each sample to be analyzed, 25.0 ± 0.2 mg of dried and ground corn stover was weighed into two separate 14 ml sterile test tubes (BD Biosciences, San Jose, CA). Two tubes were used to control for variations in fluorescence of the corn stover samples: an experimental tube to be inoculated with crp*-gfp and a control tube to be inoculated with crp*-gfp^-^. The difference in the fluorescence of these two tubes was used to determine the GFP-specific fluorescence. Then 1150 μl of 0.5% (v/v) sulfuric acid were added to each tube, and the tubes were incubated at 100°C for 1 h [14]. The tubes were allowed to cool for 15 min after incubation, after which 3850 μl of bacterial media inoculum (2 × M9 media inoculated with the appropriate bacterial culture) was added to each tube. 1 l of bacterial media inoculum contained 620 ml sterile water, 330 ml 5 × M9 salts, 6.6 ml 1 M MgSO_4_, 164.2 μl 1 M CaCl_2_, 1.7 ml thiamin at 10%, 8.3 ml kanamycin at 10 mg/ml, and 33 ml crp*-gfp or crp*-gfp^- ^liquid culture (grown overnight at 37°C in 1 × M9 media to an OD_595 _of ~0.6). In addition, 25 μl of 1:1 GC220: Multifect^® ^Xylanase (Genencor Intl.) were added to each tube. The tubes were allowed to incubate with shaking at 37°C and 225 rpm. Samples containing 100 μl of 0, 2, 4, 6, 8, 10, 12, 14, 16, or 18 mg/ml sugar at ratios of 37.5 xylose: 62.5 glucose in place of corn stover were included as positive controls. Absorbance and fluorescence were measured after 20 h of incubation by allowing the stover particles to settle in the culture tube and transferring 100 μl of the culture to a 96-well plate.

The GFP-specific fluorescence values were computed by subtracting the fluorescence from crp*-gfp^- ^cultures from crp*-gfp cultures. These values were then analyzed by ANOVA in order to characterize variation in the experiment. When variation was significant, a student's *t*-test was performed on each pair to compare means of the samples. The coefficient of variance (CV) was also computed in order to determine the variation in measurements for each genotype.

#### Hydrolysis monitoring over time

The SSC method described above was used to analyze stover samples from five corn varieties. Absorbance and fluorescence were measured every 2 h for 24 h and once at 36 h.

## Results

### Feedback inhibition of a preparation of cellulases and hemicellulases

Enzyme Multifect^® ^A-40 was strongly inhibited by the relatively low level of glucose (10 mM d-glucose). We observed 10-fold or greater reductions in glucose yield when hydrolyzing corn stover in the presence of added glucose (data not shown). This clearly demonstrated the need for an assay in which the hydrolysis products were removed from the hydrolysis reaction as they were produced.

### Characterization of the microbial system

**Figure 1:**
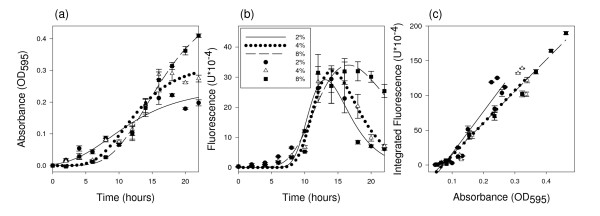
**Growth curves with different glucose concentrations**. Growth curves of *E. coli *crp*-gfp grown in modified 1 × M9 minimal media containing 2, 4, or 8% glucose. All points are the mean of four replications with error bars indicating the standard error. (a) Data was fit to curves described by the Gompertz equation. *R*^2 ^values: 2%, 0.85; 4%, 0.91; 8%, 0.97. (b) Each replication consisted of the fluorescence of a crp*-gfp^- ^culture subtracted from the fluorescence of a crp*-gfp culture. Data was fit to first derivative Gompertz equation. *R*^2 ^values: 2%, 0.80; 4%, 0.82; 8%, 0.82. (c) Mean fluorescence values from panel b were integrated and plotted against mean crp*-gfp absorbance values from panel a. Linear fit *R*^2 ^values: 2%, 0.90; 4%, 0.93; 8%, 0.98 (*n *= 4).

The objective was to establish whether it was possible to detect differences in changes in culture cell mass using GFP as a reporter and to determine the relationship between these changes in culture cell mass and GFP fluorescence. Three different concentrations of glucose were added to culture media and the absorbance and fluorescence of the cultures were measured over time. The absorbance data were fit to the Gompertz equation [15,16] which describes the normal sigmoidal growth of bacteria over time (Figure 1a). The fluorescence data, however, fit a Gompertz equation poorly, particularly late in the experiment (Figure 1b), suggesting that GFP fluorescence is not simply proportional to culture cell mass. This lack of proportionality was one key conclusion of Leveau and Lindow's modeling effort [17].

Based on the data in Figures 1a and 1b, we observed that the fluorescence signal tracked the rate of change of the optical density (OD), leading us to hypothesize that fluorescence is proportional to the instantaneous rate of change in cell culture mass, *dX/dt *(measured by monitoring culture turbidity with time) of the culture. To test this hypothesis, these data were fit to the first derivative of the Gompertz equation (Figure 1b). Our hypothesis can be stated thus: the integral of the fluorescence data over time is proportional to the cell mass of the culture. This 'integrated fluorescence' approach was taken in Figure 1c, which illustrates the high correlation (*R*^2 ^values from 0.85 to 0.97) between the integrated fluorescence and culture OD. To further characterize the relationship between initial sugar concentration and GFP-specific fluorescence, we regressed GFP-specific fluorescence at 20 h, and integrated fluorescence from 0 to 20 h, on initial glucose concentration, finding *R*^2 ^values of 0.91 and 0.98 respectively. The integrated fluorescence was the better predictor of initial sugar concentration. The 20 h GFP-specific fluorescence predicted sugar concentration fairly well (*R*^2 ^of 0.91), so this value would be useful in a high-throughput screening method where it may not be realistic to take measurements throughout the reaction. The absorbance and fluorescence data from the growth curves (Figure 1) suggest single time-point measurements could be made at any point between 16 and 22 hours of growth with similar results. The 20 h value may be indicative of the amount of sugar being metabolized at the 20 h sampling time, and may therefore be sensitive to factors such as enzyme load and biomass recalcitrance. Other sampling times need to be explored to find the most informative time, especially if different hydrolytic conditions and feedstocks are used.

**Figure 2:**
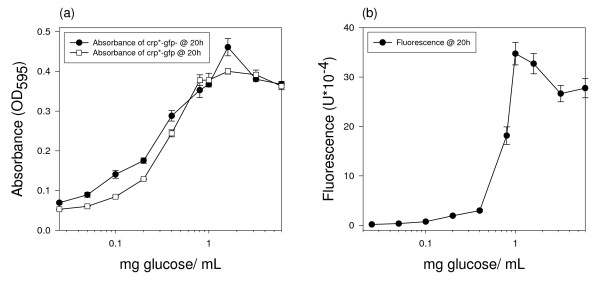
**Sensitivity and dynamic range**. *E. coli *strain crp*-gfp grown in modified 1 × M9 minimal media for 20 h with increasing amounts of glucose. (a) Each time point is the average absorbance of four replications of each *E. coli *strain. (b) Each time point is the average fluorescence of four replications of crp*-gfp cultures minus the average fluorescence of four replications of crp*-gfp^- ^cultures. All points are the mean ± s.e.

The sensitivity and dynamic range of our microbial assay were estimated in order to determine whether the microbe is useful in a corn stover hydrolysis assay. Several different concentrations of glucose were added to culture media, and the absorbance and fluorescence were measured 20 h after inoculation. As shown in Figure 2b, the sensitivity of the microbial assay was 0.4 mg glucose/ml solution, and the dynamic range was from 0.4 mg to 1.0 mg glucose/ml solution, which should be sufficient for monitoring stover hydrolysis. This dynamic range could be greatly increased by using multiple fluorescence measurements to compute an integrated fluorescence value, but we did not do this in these experiments because our purpose was to examine single time-point measurements for monitoring large numbers of samples.

**Figure 3:**
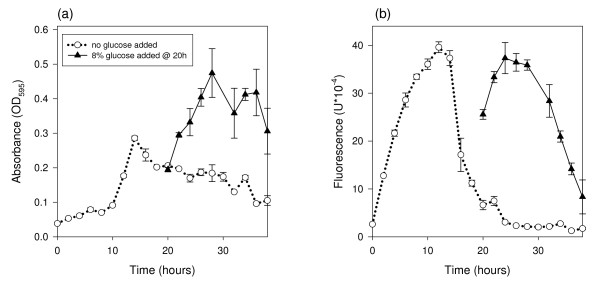
**Response to the addition of glucose**. *E. coli *strains crp*-gfp and crp*-gfp^- ^grown in modified 1 × M9 minimal media containing 2% glucose for 20 h. 8% glucose was added after 20 h to the indicated cultures. (a) Each time point is the average absorbance of three replications. (b) Each time point is the average fluorescence of three replications of crp*-gfp cultures minus the average fluorescence of three replications of crp*-gfp^- ^cultures. All points are the mean ± s.e.

We conducted two experiments to determine the response time of the microbial system to either a flux in sugar concentration or a sudden limitation in sugar during a hydrolysis reaction. The first objective was to determine how quickly the microbe responded to adding glucose to a reaction where glucose was limiting. To test this, after growing several cultures on 2 mg/ml glucose to stationary phase, we spiked half the cultures with 8 mg/ml sugar then monitored the response of the microbe. This spiking caused the absorbance and fluorescence to increase (Figure 3). As observed previously, the fluorescence level changed in proportion to the change in the cell mass with time, *dX/dt*. Within 2 h the effect of glucose addition was evident and fluorescence reached a maximum after 4 h. Because we conducted a time-course experiment, we were also able to calculate the integrated fluorescence. By plotting the glucose concentrations (2 mg/ml and 10 mg/ml) against the fluorescence units from either GFP-specific fluorescence or integrated fluorescence at 18 h, the *R*^2 ^values were 0.86 and 0.99 respectively (data not shown), confirming our hypothesis that integrated fluorescence values are the most accurate predictors of sugar concentration.

**Figure 4:**
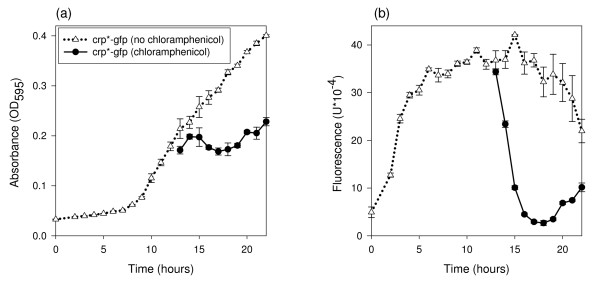
**Response to the addition of chloramphenicol**. *E. coli *strains crp*-gfp and crp*-gfp^- ^grown in modified 1 × M9 minimal media, containing 20% d-glucose, with or without chloramphenicol. Chloramphenicol added at 13 h. (a) Each time point for hours 0 to 12 is the average absorbance of six replications. Each time point for hours 13 to 23 is the average absorbance of three replications. (b) Each time point for hours 0 to 12 is the average fluorescence of six replications of crp*-gfp cultures minus the average fluorescence of six replications of crp*-gfp^- ^cultures. Each time point for hours 13 to 23 is the average fluorescence of three replications of crp*-gfp cultures minus the average fluorescence of three replications of crp*-gfp^- ^cultures. All points are the mean ± s.e.

In addition to determining how quickly the microbe can respond to an increase in sugar concentration, it is important to establish the response time to a sudden limitation in sugar. We reasoned that the response to a sudden limitation in sugar would be limited by the rate of decay of existing GFP, so we sought to determine this parameter by halting protein production in mid-log phase and determining the effect on cell density and GFP-fluorescence. This was accomplished by the addition of chloramphenicol (a bacterial translation inhibitor) to the bacterial cultures and measuring absorbance and fluorescence over time. Absorbance measurements showed that the bacteria entered stationary phase 1 h after addition of chloramphenicol, and the fluorescence decreased proportionally to the decrease in rate of growth (Figure 4). Within 1 h, GFP fluorescence decreased dramatically, reaching a minimum after 4 h.

### Application of the microbial system

Post-hydrolysis monitoring is important to show the applicability of the microbial system to screening corn stover samples for their suitability for hydrolysis. The SSC bioassay described here is a high-throughput screening method that can differentiate corn stover samples based on their sugar yield from hydrolysis. The corn stover samples chosen for this experiment were five near-isogenic lines, four of which were near-isogenic for a different *brown midrib *allele: W64A × A619 (wild type), W64A × A619 bm1, W64A × A619 bm2, W64A × A619 bm3, and W64A × A619 bm4. The *brown midrib *mutations either alter the composition or reduce the amount of lignin in the corn stover, making the stover more conducive to hydrolysis [18]. We hypothesized that the *brown midrib *lines would yield more sugar upon hydrolysis than the line without the *brown midrib *phenotype when performing endpoint hydrolysis on this set of corn stover samples. There was a significant difference in the mean GFP-specific fluorescence values for all four *brown midrib *mutants when compared with the near-isogenic line without the *brown midrib *mutation (Table 1). The *brown midrib *lines containing alleles *bm1*, *bm2*, and *bm3 *had significantly higher mean GFP-specific fluorescence values than the line containing the *bm4 *allele. It is important to note that none of the stover samples were completely hydrolyzed to available sugars. This was done intentionally in order to allow us to differentiate samples.

**Table 1:** Endpoint hydrolysis of corn stover

Corn Stover	Mean GFP-specific fluorescence (U*10^-4^)^a^	CV%^b^	Grouping^c^
W64A × A619	7.3	25.1	C
W64A × A619 bm1	19.8	19.1	A
W64A × A619 bm2	19.6	14.4	A
W64A × A619 bm3	21.7	13.8	A
W64A × A619 bm4	15.1	20.6	B

^a ^Mean GFP-specific fluorescence is the mean of the fluorescence from crp*gfp^- ^subtracted from crp*-gfp (*n *= 6).

^b ^CV% is the standard deviation divided by the mean *100 (*n *= 6).

^c ^Levels not connected by same letter are significantly different, *p *< 0.05.

**Figure 5:**
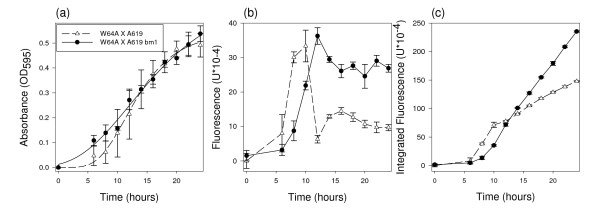
**Real-time hydrolysis of corn stover**. Simultaneous saccharification and catabolism method used to analyze corn stover samples W64A × A619 and W64A × A619 bm1. (a) Absorbance measured every 2 h for 24 h. Each point is the mean ± s.e. (*n *= 2). (b) Fluorescence measured 2 h for 24 h. Each point is the mean ± s.e. (*n *= 2). (c) Mean fluorescence values integrated and plotted over time. Each point is the mean ± s.e. (*n *= 2).

For some applications, it may be important to monitor the products of a hydrolysis reaction with our microbial system over time. For example, this may be useful for optimizing mixtures of hydrolytic enzymes. The near-isogenic hybrids W64A × A619 and W64A × A619 bm1 were analyzed using SSC and the culture OD and fluorescence were measured over time. Our hypotheses, based on the previous growth curve observations, were that instantaneous GFP-specific fluorescence would be proportional to the sugar catabolism rate (and therefore to the change in cell culture mass, *dX/dt*), and that the integral of fluorescence would therefore be proportional to the culture cell mass, *X*, which in turn is proportional to the amount of sugar consumed by the microbe. It was expected that the integrated fluorescence values would be the most accurate predictors of sugar concentrations during SSC because they are not based on a specific growth model but on the total sugar catabolized by the microbe (as shown earlier by the glucose-spiking experiment). Under this assumption, there was no reason to fit a Gompertz equation or the first derivative of the Gompertz equation to the data because neither would be expected to accurately model culture growth when sugars are being produced during culture growth. The absorbance and fluorescence data are shown in Figures 5a and 5b, respectively. The integral of fluorescence was the best indicator of sugar concentration in previous experiments and these values are presented in Figure 5c. For single time-point measurements, the difference between the corn stover with more available sugars and the corn stover with less available sugars is best determined during the time period of steady-state fluorescence (16 to 24 h), whether the GFP-specific fluorescence value or the integrated fluorescence value is used. However, it is likely that the integrated fluorescence value more accurately predicts the total amount of sugar catabolized by the microbe up to a specific time point. The W64A × A619 bm1 hybrid appeared to be over 50% more digestible than then W64A × A619 hybrid, based upon the 20 h integrated fluorescence value (230 vs. 150 Units × 10^-4^, Figure 5c).

## Discussion

### Feedback inhibition of a preparation of cellulases and hemicellulases

We have shown that Multifect^® ^A-40, a commercially available hydrolytic enzyme preparation, is feedback inhibited, which means that assays involving quantitation of hydrolysis products at the end of a reaction may not be very useful for evaluating sources of biomass.

### Characterization of the microbial system

We elected to develop a fluorescent reporter system to aid in characterizing culture growth. In such a system, it is important to consider that inner filter effects [19] can influence the observed fluorescence of a culture system. These effects can be classified into two sources. First, they can be fluorescent components of the culture with emission spectra that overlap the emission spectrum of the analyte of interest. In analysis of corn stover, we control these effects by subtracting the fluorescence value of a control sample that is identical to the test sample, except the *GFP *gene has been disabled by mutation. The second source of inner filter effects is components of the culture that are non-fluorescent and interfere with the excitation and/or emission light. In our experiments, the ground corn stover particles or the bacterial cells themselves could contribute to this effect. We minimize the effect of the stover particles by allowing them to settle prior to transferring a portion of the culture to a microtiter plate for analysis. The approximate impact of bacterial cells themselves is reflected in the culture turbidity. It is probably not appropriate to compare samples that differ widely in culture turbidity based on their fluorescence values. We did not observe extreme differences in turbidity of the samples we analyzed; however, if observed, these differences in turbidity may form the basis of another method of interpreting the assay.

Based on modeling by Leveau and Lindow [17] we expected to see the fluorescence levels of the liquid cultures decrease over time. This was the case as shown in Figure 1 by the growth curves with different glucose concentrations. GFP fluorescence can be limited by carbon as well as by nitrogen and oxygen [20], but we have determined through experimentation that nitrogen and oxygen are not limiting (data not shown). One possible explanation for the observed decrease in fluorescence in the later stages of growth is GFP protein denaturation. This would be problematic if experimental treatments influenced the rate of denaturation, and necessitate caution when comparing treatments likely to have an impact on GFP denaturation. Differences in GFP spectral properties have been characterized under conditions of varying pH, temperature, ionic strength and protein concentration [21]. These spectral changes were reversible, leading the authors to conclude that the GFP chromophore is conformationally flexible. In the experiments presented here, factors known to influence GFP stability were not varied within an experiment and therefore different rates of GFP denaturation probably had minimal influence on the outcome. It is possible that GFP is being actively degraded for use as a carbon source; however, it is not clear if or how this possibility impacts the relationship between fluorescence and growth rate. We used the Gompertz equation to describe the trend of cell growth over time and the first derivative of the Gompertz equation to describe the trend of fluorescence over time. This showed that the rate of change in cell culture mass with time, *dX/dt *(described by the first derivative of the Gompertz equation) was proportional to fluorescence at any point in time. We also showed that a higher amount of sugar in solution will cause the cells to stay in the log growth phase, and therefore have higher fluorescence, for a longer period of time than when sugar levels are lower. (This is assuming these sugar solutions are within the dynamic range of the microbial assay.) These data support our revised hypothesis that instantaneous fluorescence is proportional to the sugar catabolism rate. Based on the growth curves with different glucose concentrations, it is best to measure fluorescence between 16 and 22 h of growth when the *E. coli *are limited in carbon and the rate of growth is most different. As we observed a high correlation (*R*^2 ^of 0.98) between sugar concentrations and integrated fluorescence at 20 h, we hypothesize that the integral of fluorescence is proportional to the amount of sugar consumed by the microbe. We verified this in the glucose spiking experiment.

The sensitivity and dynamic range of the microbial assay allows for the detection of sugar in the hydrolysis reactions performed. We were able to observe significant differences between the amounts of sugar produced in the corn stover hydrolysis reactions with only 25 mg of sample. The amount of sugar produced by the hydrolysis reaction is within the dynamic range of the microbial assay.

### Application of the microbial system

The data presented in this paper establish a clear relationship between fluorescence and culture growth rate. To meet the objective of using this system to monitor sugars present in biomass and/or produced in hydrolytic reactions, we must assume that culture cell mass is proportional to the amount of sugar available to the culture. While this was the case in cultures containing purified sugars, this may not be the case when the sugars are provided from biomass. Compounds present in biomass or released by its hydrolysis that support or inhibit microbial growth would interfere with this assay. Further experiments to quantify the magnitude of growth promotion or inhibition effects of biomass would increase the utility of this assay.

Our data suggest that post hydrolysis monitoring (that is, measuring fluorescence levels at the end of the hydrolysis reaction) is feasible. Our evaluation of low lignin *brown midrib *mutants using this approach showed that they yield a significantly higher amount of available sugars per unit mass than a genotype with wild-type lignin levels. This result is consistent with the large body of data suggesting the low lignin genotypes of both corn and sorghum are more readily hydrolyzed and digestible than their normal lignin counterparts [22-27].

In the real-time corn stover hydrolysis, shown in Figures 5a and 5b, during the first 16 h, the fluorescence appears to be proportional to the change in cell culture mass with time, *dX/dt*. After 16 h, the fluorescence appears to be proportional to the cell density. If the values of the absorbance and fluorescence are predicted between 24 and 36 h and the integral of the fluorescence versus absorbance is plotted, there is a clear deviation from a linear trend at an OD_595 _of ~0.5 where the predicted values cause the slope to increase dramatically (data not shown). This deviation is most likely due to a new and steady supply of sugar to the bacteria provided by the hydrolytic enzymes. Based on the data shown in Figure 5, we predict that this constant supply of sugar is due to the equilibrium between sugar consumption by the bacteria and sugar production by the hydrolytic enzymes, which is controlled by feedback inhibition of the hydrolytic enzymes in high sugar concentrations. This constant supply of sugar leads to a constant change cell culture mass with time, which in turn leads to constant levels of GFP fluorescence. This explains why these data do not fit a Gompertz equation, which assumes required nutrients are in excess.

From the data presented here, we have hypothesized that during growth the level of sugars starts high and decreases during the course of the reaction until the sugar levels limit growth. In contrast, when the sugar required for the growth of the microbe is provided by a hydrolysis reaction such as in the SSC method, we hypothesize that a certain amount of sugar is present in solution thereby inhibiting enzymatic hydrolysis of the corn stover until the microbes can deplete the sugars enough for the enzymes to regain function. From the point in time when the enzymes regain function, the hydrolysis proceeds in equilibrium with bacterial growth until the feedstock becomes depleted and the hydrolysis reaction slows. For example, a stover sample more conducive to hydrolysis would allow for sugar to be released by the enzyme into solution more rapidly, therefore supporting a higher steady-state level of GFP. This would give a higher amount of steady-state fluorescence. A sample less conducive to hydrolysis would release the sugars more slowly causing lower steady-state GFP levels.

It may be possible to develop a method that predicts the suitability of biomass for enzymatic hydrolysis using the microbial system described here. This would involve using the microbial system to monitor a small-scale version of the hydrolytic process of interest. The small-scale method should be designed to emulate the large-scale process as closely as possible. For example, as described here, the results of our process will be influenced by the presence of soluble sugars. If the hydrolytic process of interest does not capture the soluble sugars from the biomass, then it may be appropriate to wash the biomass prior to hydrolysis in the small-scale system.

## Conclusion

We characterized growth of an *E. coli *strain designed to report sugar levels in lignocellulosic biomass hydrolysis reactions. The sugar-sensing system described here overcomes product inhibition of hydrolytic enzymes by removing the sugars from solution as the culture grows. This allows the enzymes to hydrolyze the biomass further and gives us the ability to differentiate samples based on their suitability for hydrolysis. Because the microbe expresses GFP constitutively, it allows the use of fluorescence as a measure of sugar concentration when the solution is too turbid to measure absorbance accurately or when other compounds interfere with this culture density measurement.

We found that instantaneous fluorescence is proportional to the change in culture cell mass with time, *dX/dt*, based on growth curves with different glucose concentrations. We hypothesized that the integral of fluorescence is proportional to the amount of sugar consumed by the microbe, and we verified this in a glucose-spiking experiment. By using our fluorescence-based method to monitor sugars released from hydrolysis reactions with corn stover samples, we found that post-hydrolysis monitoring could be used as a high-throughput screening method to determine available sugars and, in turn, to predict ethanol production potential in different feedstocks. Also, it may be possible to use the real-time hydrolysis of corn stover method to compare different hydrolytic enzymes by using the same feedstock and monitoring the release of sugars by measuring fluorescence during the course of the reaction.

## Competing interests

The authors declare that they have no competing interests.

## Authors' contributions

LJH carried out all experiments and analyses in the studies and drafted the manuscript. JGC and AJL selected, produced and collected corn stover samples and prepared them for analysis. DRR and RPA interpreted and modeled the time-sequence data and significantly edited the manuscript. RPA provided project integration and coordination as PI of the 'Integrated Feedstock Supply Systems for Corn Stover Biomas' grant. MPS conceived of the study, participated in its design and coordination and helped to draft the manuscript. All authors read and approved the final manuscript.

## References

[B1] Hahn-Hagerdal B, Galbe M, Gorwa-Grauslund MF, Liden G, Zacchi G (2006). Bio-ethanol – the fuel of tomorrow from the residues of today. Trends Biotechnol.

[B2] Lynd LR, Wyman CE, Gerngross TU (1999). Biocommodity engineering. Biotechnol Prog.

[B3] Wright JD, Wyman CE, Grohmann K (1988). Simultaneous saccharification and fermentation of lignocellulose: process evaluation. Appl Biochem Biotechnol.

[B4] Dowe N, McMillan J (1995). SSF experimental protocols: cellulosic biomass hydrolysis and fermentation. Protocol LAP008.

[B5] Weimer PJ, Dien BS, Springer TL, Vogel KP (2005). In vitro gas production as a surrogate measure of the fermentability of cellulosic biomass to ethanol. Appl Microbiol Biotechnol.

[B6] Kimura A, Robyt JF (1995). Reaction of enzymes with starch granules: Kinetics and products of the reaction with glucoamylase. Carbohydr Res.

[B7] Fox JD, Robyt JF (1991). Miniaturization of three carbohydrate analyses using a microsample plate reader. Anal Biochem.

[B8] Lidgren L, Lija O, Krook M, Kriz D (2006). Automatic fermentation control based on a real-time in situ SIRE^® ^biosensor regulated glucose feed. Biosens Bioelectron.

[B9] Sabourin D, Beckwith J (1975). Deletion of the *Escherichia coli crp *gene. J Bacteriol.

[B10] Axtell CA, Beattie GA (2002). Construction and characterization of a proU-gfp transcriptional fusion that measures water availability in a microbial habitat. Appl Environ Microbiol.

[B11] Miller WG, Lindow SE (1997). An improved GFP cloning cassette designed for prokaryotic transcriptional fusions. Gene.

[B12] Heim R, Cubitt AB, Tsien RY (1995). Improved green fluorescence. Nature.

[B13] Sambrook J, Russell DW (2001). Molecular Cloning: A Laboratory Manual.

[B14] Mohagheghi A, Dowe N, Schell D, Chou Y-C, Eddy C, Zhang M (2004). Performance of a newly developed integrant of *Zymomonas mobilis *for ethanol production on corn stover hydrolysate. Biotechnol Lett.

[B15] Gompertz B (1825). On the nature of the function expressive of the law of human mortality, and on a new mode of determining the value of life contingencies. Philos Trans R Soc London.

[B16] Zwietering MH, Jongenburger I, Rombouts FM, Riet KVT (1990). Modeling of the bacterial growth curve. Appl Environ Microbiol.

[B17] Leveau JHJ, Lindow SE (2001). Predictive and interpretive simulation of green fluorescent protein expression in reporter bacteria. J Bacteriol.

[B18] Marita JM, Vermerris W, Ralph J, Hatfield RD (2003). Variations in the cell wall composition of maize brown midrib mutants. J Agric Food Chem.

[B19] Srinivas SP, Mutharasan R (1987). Inner filter effects and their interferences in the interpretation of culture fluorescence. Biotechnol Bioeng.

[B20] Cubitt AB, Heim R, Adams SR, Boyd AE, Gross LA, Tsien RY (1995). Understanding, improving and using green fluorescent proteins. Trends Biochem Sci.

[B21] Ward WW, Prentice HJ, Roth AF, Cody CW, Reeves SC (1982). Spectral perturbations of the aequorea green fluorescent protein. Photochem Photobiol.

[B22] Barnes RF, Muller LD, Bauman LF, Colenbrander VF (1971). *In vitro *dry matter disappearance of brown midrib mutants of maize (*Zea mays *L.). J Anim Sci.

[B23] Lechtenberg VL, Muller LD, Bauman LF, Rhykerd CL, Barnes RF (1972). Laboratory and *in vitro *evaluation of inbred and F_2 _populations of brown midrib mutants of *Zea Mays *L. Agron J.

[B24] Muller LD, Barnes RF, Bauman LF, Colenbrander VF (1971). Variations in lignin and other structural components of brown midrib mutants of maize. Crop Sci.

[B25] Bucholtz DL, Cantrell RP, Axtell JD, Lechtenberg VL (1980). Lignin biochemistry of normal and brown midrib mutant sorghum. J Agric Food Chem.

[B26] Fritz JO, Cantrell RP, Lechtenberg VL, Axtell JD, Hertell JM (1981). Brown midrib mutants in sudangrass and grain sorghum. Crop Sci.

[B27] Porter KS, Axtell JD, Lechtenberg VL, Colenbrander VF (1978). Phenotype, fiber composition, and *in vitro *dry matter disappearance of chemically induced brown midrib (bmr) mutants of sorghum. Crop Sci.

